# A quantum inspired machine learning approach for multimodal Parkinson’s disease screening

**DOI:** 10.1038/s41598-025-95315-0

**Published:** 2025-04-04

**Authors:** Diya Vatsavai, Anya Iyer, Ashwin A. Nair

**Affiliations:** 1 Valley Christian High School, San Jose, CA USA; 2 Dougherty Valley High School, San Ramon, CA USA; 3 UC Davis Graduate School of Management, Davis, CA USA

**Keywords:** Computational models, Machine learning, Predictive medicine

## Abstract

Parkinson’s disease, currently the fastest-growing neurodegenerative disorder globally, has seen a 50% increase in cases within just two years. As disease progression impairs speech, memory, and motor functions over time, early diagnosis is crucial for preserving patients’ quality of life. Although machine-learning-based detection has shown promise for detecting Parkinson’s disease, most studies rely on a single feature for classification and can be error-prone due to the variability of symptoms between patients. To address this limitation we utilized the mPower dataset, which includes 150,000 samples across four key biomarkers: voice, gait, tapping, and demographic data. From these measurements, we extracted 64 features and trained a baseline Random Forest model to select the features above the 80th percentile. For classification, we designed a simulatable quantum support vector machine (qSVM) that detects high-dimensional patterns, leveraging recent advancements in quantum machine learning. With this novel and simulatable architecture that can be run on *standard* hardware rather than resource-intensive quantum computers, our model achieves an accuracy of 90%, F-1 score of 0.90, and an AUC of 0.98—surpassing benchmark models. Utilizing an innovative classification framework built on a diverse set of features, our model offers a pathway for accessible global Parkinson’s screening.

**Supplementary Information:**

The online version contains supplementary material available at 10.1038/s41598-025-95315-0.

## Introduction

Parkinson’s disease (PD) is a progressive neurodegenerative disorder that gradually compromises neuronal function, resulting in motor and nonmotor impairments^1^. Central to PD pathology is the degeneration of dopaminergic neurons within the substantia nigra, a critical region for dopamine synthesis. An essential neurotransmitter deficiency arises as these neurons lose their dopamine-producing capacity, leading to hallmark symptoms such as bradykinesia, hypokinetic dysarthria, resting tremor, and muscular rigidity^2^. Over the past 25 years, PD prevalence has doubled, and related deaths have increased by more than 100% since 2000^3^. These trends highlight an urgent need for effective disease detection and intervention.

Current detection methods, like DAT and PET scans, are not PD-specific, often leading to imprecise results^4^. These diagnostic approaches also require expensive, specialized equipment and medical expertise, costing the U.S. around $14 billion annually^5^. As the fastest-growing neurological condition globally, PD urgently calls for a low-cost, high-accuracy screening solution^6^.

Despite recent improvements in clinical diagnostic criteria, accurately identifying PD remains difficult due to the overlap of symptoms with other neurodegenerative conditions and the normal aging process. Diagnoses based on a single or limited set of clinical features often lead to inconclusive results. Research on specific biomarkers has helped improve PD detection. In this study, we focus on vocal biomarkers, gait indicators, demographic data, and tapping metrics. Studies show that voice serves as a useful PD indicator. Notably, voice has proven particularly informative: Ma et al.^7^ found that 70–90% of individuals with PD exhibit varying degrees of vocal impairment, and up to 78% of those in early disease stages show noticeable changes^8^. Common vocal impairments associated with PD include pitch variations, decreased volume, unclear articulation, and an unstable voice. Recognizing the diagnostic potential of these speech impairments, we focus on analyzing specific vocal features associated with these changes, including volume, pitch, jitter, shimmer, and breathiness.

Gait serves as another characteristic marker of PD. Bradykinesia disrupts gait, causing both episodic and continuous disturbances^9^. Episodic disturbances include start hesitation and freezing of gait, while continuous disturbances reflect inconsistent walking patterns. These gait impairments significantly impact PD patients, with studies showing that 45-68% experience falls annually, and 50-86% fall recurrently^10^. Although gait disturbances aid in diagnosis, they also represent one of PD’s most physically deteriorating symptoms, leading to severe injuries and heightening patients’ fears of daily activities.

In addition to clinical markers such as voice and gait impairments, demographic factors—particularly age—play a critical role in detecting and understanding Parkinson’s disease (PD). Data from the Parkinson’s Foundation reveal a pronounced increase in PD incidence among individuals aged 65 and older, underscoring the importance of age-related vulnerability as an early diagnostic cue. This demographic insight not only aids in identifying higher-risk populations but also encourages more timely interventions aimed at slowing disease progression, and thus, it is included in our analysis.

Finger-tapping measurements, which serve as a proxy for bradykinesia, have proven useful yet remain controversial for PD diagnosis. Bradykinesia, a primary motor symptom of PD, manifests as slowness and halting in movement^11^. However, clinical evaluations of bradykinesia often rely on visual judgment^12^, which varies widely between clinicians. Moreover, the limited number of published studies on finger-tapping also lack robust interrater reliability estimates, emphasizing the lack of standardization. Nonetheless, some studies indicate that finger-to-thumb tapping tests correlate with lower overall UPDRS (Unified Parkinson’s Disease Rating Scale) scores in PD patients^13^. Although the UPDRS offers a standardized method for assessing PD motor severity, it cannot fully address the issues of subjectivity and interrater variability.

Multimodal data integration presents a significant challenge in PD diagnosis due to the complex interrelationships between vocal, motor, and demographic features. Traditional machine learning models often struggle when fusing disparate data types efficiently, leading to suboptimal classification performance. Each data modality exhibits distinct characteristics—vocal biomarkers involve temporal and spectral features, gait data requires spatiotemporal analysis, and demographic data adds categorical information—making it difficult for conventional models to construct a holistic understanding of PD-related patterns. Additionally, feature imbalances across these modalities can lead to model bias, reducing reliability and generalizability. As a result, a robust diagnostic system must effectively capture nonlinear relationships while mitigating the impact of missing or noisy data, ensuring comprehensive and precise predictions.

Machine learning methods have seen extensive adoption in healthcare, influencing areas that range from advanced image classification to broader public health policy^14–16^. For instance, a systematic review of AI applications in urology cancer^15^ found that convolutional neural networks (CNNs) achieved the highest detection accuracy (77–95%) for prostate, bladder, and kidney cancers. These findings highlight how AI-driven medical diagnostics, particularly those leveraging deep learning and multi-feature integration, can improve accuracy and clinical decision-making. However, just as with PD research, the review emphasized the need for more extensive, high-quality datasets to enhance the real-world clinical performance of AI models. Recent studies have explored the potential of machine learning algorithms to predict the occurrence of PD. However, most existing approaches rely on a single feature for the basis of prediction as rather than incorporating multiple data modalities, resulting in performance metrics ranging from 60 to 85%, which are generally unsuitable for clinical applications^17–20^. When it comes to diagnosis based on audio, convolutional neural networks (CNNs) have been applied to PD voice recordings, which are converted into spectrogram images for model classification. Many of these single-feature studies are built on private, homogenous datasets with minimal data points and unbalanced samples^21^. This can severely affect the reproducibility of the study as well as model generalizability due to lack of data. In addition, a substantial portion of studies relies on voice data obtained from speakers of a single language, which can introduce bias and further limit the generalizability of classification findings^22^.

To overcome the discussed limitations, our study’s objective is to provide a multimodal diagnostic framework based on vocal data, gait tracking, tapping, and demographic information to generate a comprehensive prediction of PD. Additionally, using automated detection removes the human subjectivity that arises with visual analysis of finger-tapping. Leveraging a large, publicly available dataset of over 150,000 samples, our approach ensures robustness and mitigates geographic bias. Importantly, we utilize the universal syllable “ahh” for 10 s to eliminate linguistic or accent-based confounding factors in vocal data. We utilize a custom quantum-assisted Support Vector Machine (qSVM) classifier exhibiting high performance even in classical simulations, removing the dependence on computationally expensive quantum hardware. qSVMs leverage the high-dimensional spaces of quantum Hilbert space to capture complex, nonlinear relationships that conventional methods might overlook. This enriched representation often translates into higher classification accuracy, better generalization, and potentially more efficient computations. As a result, qSVMs can outperform traditional methods, particularly in challenging tasks that involve large, heterogeneous datasets like mPower. Moreover, the quantum kernel can be simulated on standard hardware rather than relying on resource-intensive quantum computers, enabling broader access and practical implementation for large-scale clinical or research settings. Using this model, we outperform standard machine learning techniques, state-of-the-art deep learning approaches, and commonly offered qSVM architectures with an accuracy of 90% and an ROC/AUC score of 0.98. Moreover, these results derive from a diverse dataset representing participants with varied gender, age, racial, and educational backgrounds, underscoring its potential for clinical applications globally.

## Results

### Data description

We utilized the mPower public research portal, which contains measurements from over 6,000 participants—both healthy and those affected by Parkinson’s^23^. The dataset is available under protected access to certified researchers. The data includes common Parkinson’s disease biomarkers: demographic information, such as age, gender, and smoking history, as well as voice recordings, tapping measurements, and gait tracking, all recorded through a smartphone app. We restricted our analysis to participants who completed all the different tests (voice, tapping, gait) measured in the mPower dataset. Since many participants completed multiple iterations of the same test, we randomly selected a single trial per activity per participant to mitigate potential biases favoring those with repeated trials. We do so because, including multiple trials from the same participant could inadvertently skew model performance by overrepresenting that individual’s characteristics in the dataset. This risk of overrepresentation would make the model overly tailored to participants with more trials, diminishing its generalizability to broader patient populations^24^. For model training and testing, we focused on 194 participants who completed the voice, gait, and tapping tests. This subset stands out for its diversity, including male and female participants who identified as Caucasian, African American, Hispanic, East Asian, South Asian, and mixed race. The subset al.so represents a range of educational backgrounds, with 35% of participants not holding a four-year college degree. We divided these 194 samples into 164 for training and 30 for testing, ensuring a balanced representation of both Parkinson’s-affected and healthy individuals.

### Feature selection

**Fig. 1:**
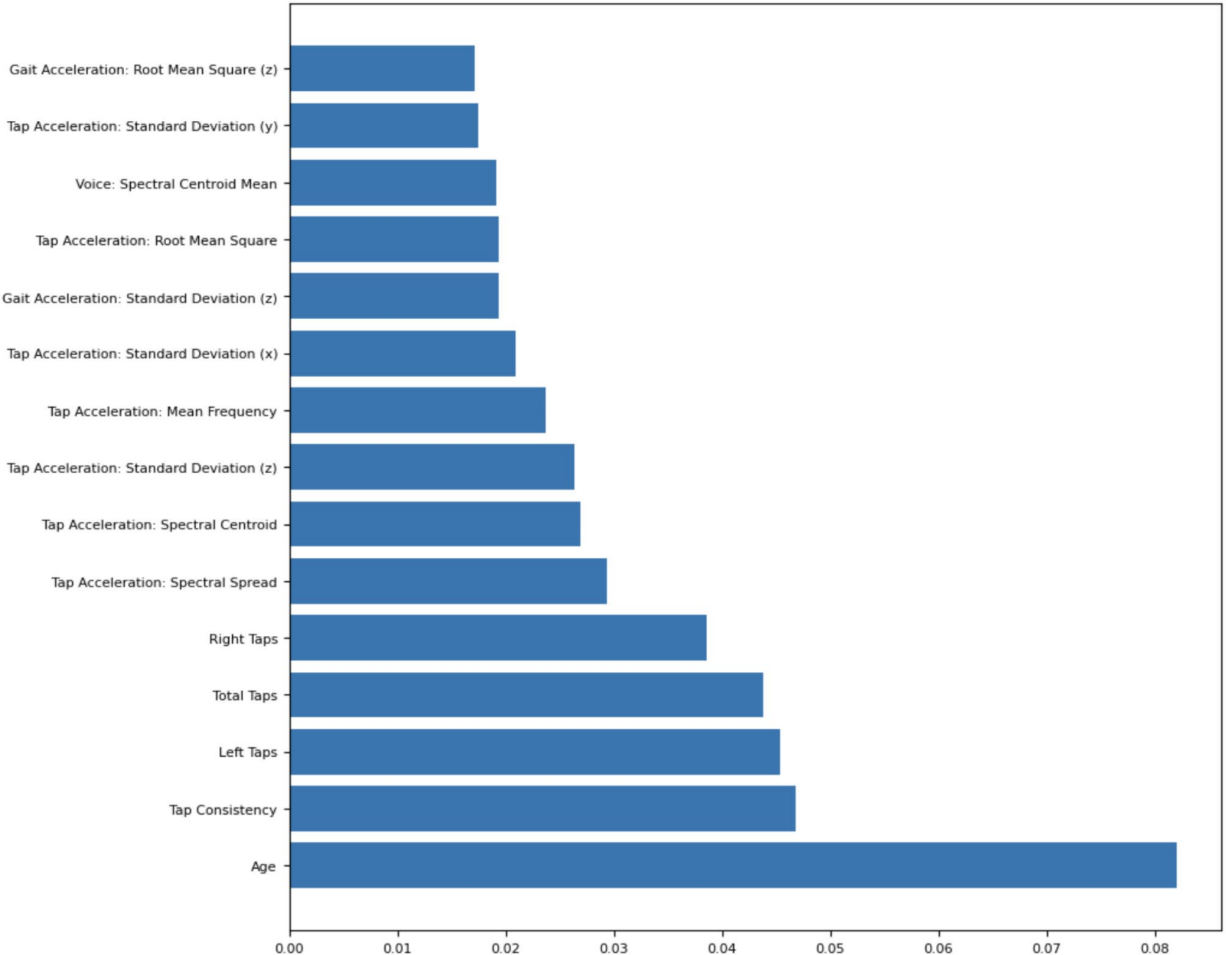
Feature importance values for features above the 80th percentile in a baseline random forest model. This figure displays the feature importance values for variables ranking above the 80th percentile in a baseline random forest model. The feature importance metric quantifies each variable’s contribution to the model’s ability to predict outcomes. Age emerges as the most significant feature, with a markedly higher importance score compared to others. This indicates its dominant role in the predictive model, potentially serving as a key demographic indicator for the task at hand. Following Age, features related to motor activity, particularly tapping metrics, demonstrate high importance. These include Tap Consistency, Left Taps, Total Taps, and Right Taps, which collectively reflect fine motor coordination and variability in tapping behavior. A notable inclusion is Voice: Spectral Centroid Mean, reflecting the role of vocal biomarkers in the analysis. Figure was generated using version 3.9.2 of the Python package Matplotlib (https://pypi.org/project/matplotlib/).

Using this dataset, we extracted 64 voice, gait, tapping, and demographic features for each of the 194 participants, balanced between healthy individuals and those with Parkinson’s. Each data modality (voice, gait, tapping, demographics) was preprocessed individually to ensure modality-specific feature extraction and noise reduction. For gait and tapping data, we extracted both time-domain metrics (e.g., root mean square, standard deviation, tapping counts) and frequency-domain features (e.g., spectral centroid, spectral spread) to capture Parkinson’s-related tremors. Vocal data consisted of 10-second “ahh” recordings, from which we derived pitch, volume, breathiness, and reduced-dimensional MFCCs, while demographic information included age, smoking history, and gender. Feature correlation was managed through random forest–based importance weighting. Additional details on feature extraction appear in the methods section. We normalized the dataframe using Scikit Learn’s StandardScaler to ensure a consistent magnitude for each feature. Next, we trained a baseline Random Forest model to identify the top-performing features for the final qSVM model, selecting features with importance values above the 80th percentile^25^ (Fig. 1).

Among the demographic features, age proved to be the most significant, consistent with extensive research illustrating a heightened risk of neurological disorders in older populations. In the voice analysis, the spectral centroid mean was the best predictor of Parkinson’s disease. This feature refers to the “center of mass” of a voice signal and often corresponds to how sharp or muffled the sound is, corresponding to the vocal changes observed in PD. Regarding the gait features, the root mean square and standard deviation of acceleration in the z direction had the highest feature importance. The root mean square, corresponding to the magnitude of acceleration, shows how forcefully the participants moved up and down when walking, and the standard deviation shows the variability of vertical acceleration, corresponding to “shaky motion”, a hallmark of Parkinson’s disease.

For the raw tapping information, the number of left taps, right taps, and total taps quantify how many times the participants tapped their screen in 20 s, providing a measure of their dexterity. Meanwhile, tapping consistency quantified by the standard deviation of the time between taps, shows whether they kept a consistent pace throughout the test. In addition to the raw tapping information, the tapping acceleration measurements from the participants’ smartphones were also significant. Among these features, the root mean square, or magnitude of acceleration, as well as the standard deviations of accelerations in each direction, captures abrupt movements characteristic of PD. Then, in the frequency domain, the average frequency and spectral centroid reflect the smoothness and consistency of tapping acceleration. Finally, the spectral spread of tapping acceleration serves as an additional indicator of the erraticness of the tapping motion, detecting tremors.

For input into the proposed qSVM model, which is highly sensitive to feature ordering and magnitude^26^, we multiplied each feature by its importance. We sorted them accordingly to emphasize the significance of higher-performing features. We then scaled all features by a factor of 10 to ensure that all features had a magnitude close to 1, enhancing the model’s ability to process the data effectively.

### Model architecture

We used Quantum Support Vector Machines (qSVMs) due to their capacity for accurately classifying high-dimensional datasets, capturing subtle patterns that might otherwise go unnoticed, similar to those in the mPower data. Quantum SVM (qSVM) models can access high-dimensional quantum Hilbert spaces, allowing them to encode complex relationships more effectively than standard classification models. This enhanced representation often translates into higher classification accuracy, improved generalization, and more efficient computations. As a result, qSVMs frequently outperform conventional SVM methods, especially for intricate classification tasks^27^. For the mPower data, which includes diverse and complex biomarkers like voice, gait, tapping, and demographic features, qSVMs can leverage quantum feature mapping to capture subtle, non-linear interactions between these heterogeneous variables. Researchers have increasingly applied qSVMs in clinical diagnosis^28^. However, quantum computing in the current noisy-intermediate scale quantum (NISQ) era remains costly, time-intensive, and error-prone^29^. To address these challenges, our study introduces a quantum-inspired kernel architecture that we simulate on classical hardware, while still outperforming traditional models. However, unlike many current qSVM kernels, our model does not rely on entanglement, which is challenging to simulate classically; instead, it uses dynamic angle embedding^30^ to capture complex data patterns without the overhead of full quantum computation.

### Evaluation and comparative analysis

Once we constructed the custom qSVM architecture, we trained the model on our dataframe of 194 samples. Afterward, we compared the accuracy, ROC/AUC score, recall/sensitivity, specificity, and precision to current state-of-the-art models in the field to demonstrate the viability of our approach. These benchmarks collectively provide a comprehensive evaluation of the model’s diagnostic capabilities: accuracy measures overall correctness, ROC/AUC balances true- and false-positive rates, recall (sensitivity) quantifies the ability to detect actual PD cases, specificity ensures minimal false alarms among healthy individuals, and precision assesses correctness among predicted positives. Benchmarking against established state-of-the-art methods, we provide evidence of the proposed model’s viability and robustness across multiple clinical performance criteria. These included architectures that have been explored for medical applications in the past, such as neural networks, SVM and qSVM models, and random forests. The comparative results are displayed in Table 1.

**Table 1:** Performance of various models across accuracy, ROC/AUC, precision, and recall.

Model	Accuracy	ROC/AUC	F1 Score	Precision	Recall/Sensitivity	Specificity
Proposed	0.90	0.98	0.91	0.94	0.89	0.91
Classical ML						
Linear SVM	0.77	0.97	0.79	0.93	0.68	0.91
Polynomic SVM	0.70	0.92	0.69	1.00	0.53	1.00
RBF SVM	0.70	0.89	0.71	0.92	0.58	0.91
Random Forest	0.60	0.94	0.54	1.00	0.37	1.00
Gradient Boost	0.63	0.89	0.59	1.00	0.42	1.00
Naive Bayes	0.63	0.92	0.62	0.90	0.47	0.91
Logistic Regression	0.73	0.97	0.73	1.00	0.58	1.00
KNN^31^	0.67	0.94	0.67	0.91	0.53	0.91
CNN^22^	0.53	0.71	0.50	0.78	0.37	0.82
FNN^32^	0.67	0.94	0.67	0.91	0.53	0.91
DNN^33^	0.77	0.92	0.79	0.93	0.68	0.91
Quantum SVM						
Z Feature Map	0.87	0.86	0.90	0.90	0.90	0.80
ZZ Feature Map	0.67	0.68	0.80	0.67	1.00	0.00

This table highlights the performance of various machine learning models across metrics such as accuracy, ROC/AUC, F1 score, precision, and recall. The Proposed Model stands out as the best-performing model, achieving an accuracy, F1 score, and recall of 0.90, along with an ROC/AUC of 0.98. Among the classical machine learning models, the Linear SVM and Logistic Regression performed with accuracies of 0.77 and 0.73, and ROC/AUC values of 0.96 and 0.97, respectively. In contrast, models like Random Forest and Naive Bayes showed lower accuracies of 0.60 and 0.63. Neural network-based approaches varied in performance, with the DNN achieving results of 0.80 accuracy and 0.93 ROC/AUC, outperforming other deep learning models such as the FNN and CNN. Lastly, Quantum SVM models, particularly with the Z Feature Map, demonstrated strong performance, achieving 0.87 accuracy and competitive overall metrics.

**Table 2:** This confusion matrix summarizes the classification performance of the proposed model on the 30 test subjects.

	Predicted positive	Predicted negative	Total
Actual positive	17	2	19
Actual negative	1	10	11
Total	18	12	30

Each cell represents the count of subjects according to their actual and predicted classifications.

**Fig. 2:**
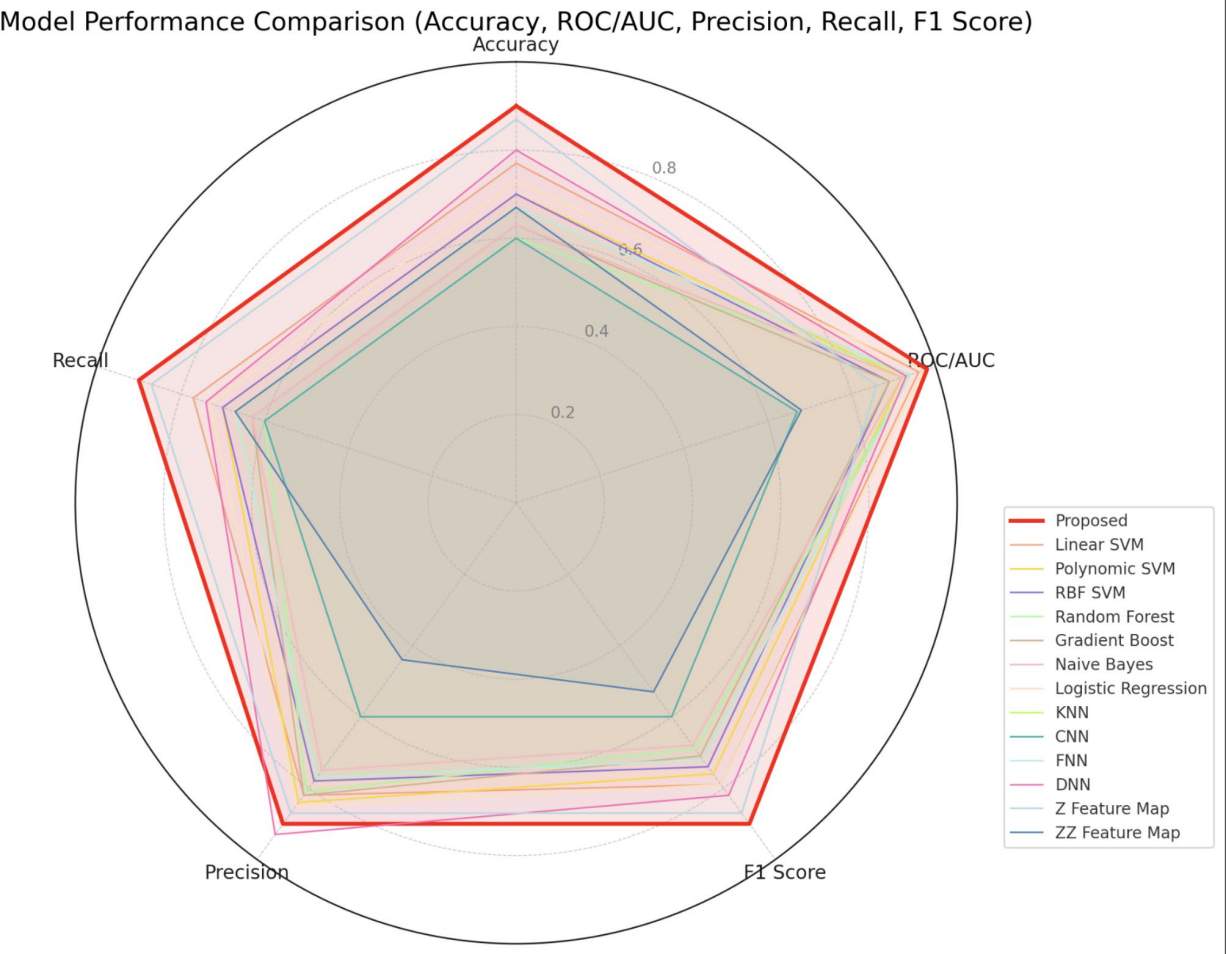
Radar chart for proposed model and benchmark comparison across accuracy, precision, recall, F1 score and ROC/AUC score. This figure presents a radar chart comparing the performance of the proposed model and various benchmark machine learning algorithms across five metrics: accuracy, ROC/AUC, precision, recall, and F1 score. The proposed model, represented in red, demonstrates consistently high values across all metrics. In comparison, models such as Linear SVM and Logistic Regression show moderate performance with balanced scores, while Naive Bayes and Random Forest display lower performance levels, as indicated by their proximity to the center of the chart. Neural network-based approaches, including DNN and CNN, exhibit competitive performance, with DNN achieving slightly higher precision and recall. Quantum machine learning models, such as those utilizing the Z Feature Map, perform well in certain metrics like accuracy and F1 score, though their overall scores remain distinct from the proposed model. The radar chart provides a comprehensive visual comparison of the models, highlighting variations in performance across the selected evaluation metrics. Figure was generated using version 3.9.2 of the Python package Matplotlib (https://pypi.org/project/matplotlib/).

Due to the extensive feature set and the high data complexity, models that incorporated strong overfitting protections generally performed better (Table 1). The corresponding confusion matrix for the proposed model is presented in Table 2. Among the classical algorithms, logistic regression and the linear SVM demonstrated the best performance. By contrast, complex neural networks tended to overfit, reducing their accuracy. Among alternative qSVMs, the entanglement-heavy ZZ feature map performed poorly, likely because classical simulators cannot emulate entanglement accurately. The Z feature map without entanglement performed better but lacked the complexity necessary to capture the full dimensionality of the data, as reflected in its lower metrics. The proposed kernel, by using quantum rotation gates to encode features into a complex quantum state without requiring entanglement, achieved the highest performance across accuracy, ROC/AUC score and F1 score (Fig. 2), emphasizing its potential as a baseline for future clinical applications.

We incorporated statistical tests including McNemar’s tests on classification outcomes, which yielded p-values ranging from 0.00049 to 0.0625, indicating that our qSVM model significantly outperforms classical and deep learning benchmarks. The low p-values (mostly < 0.05) confirm that the performance differences are unlikely due to chance, reinforcing the robustness of our model’s 90% accuracy. This demonstrates that qSVM effectively captures complex, nonlinear relationships in the data, improving classification performance over traditional methods. The results validate our approach, showing the model’s superior predictive power.

### Feature importance in classification

**Fig. 3:**
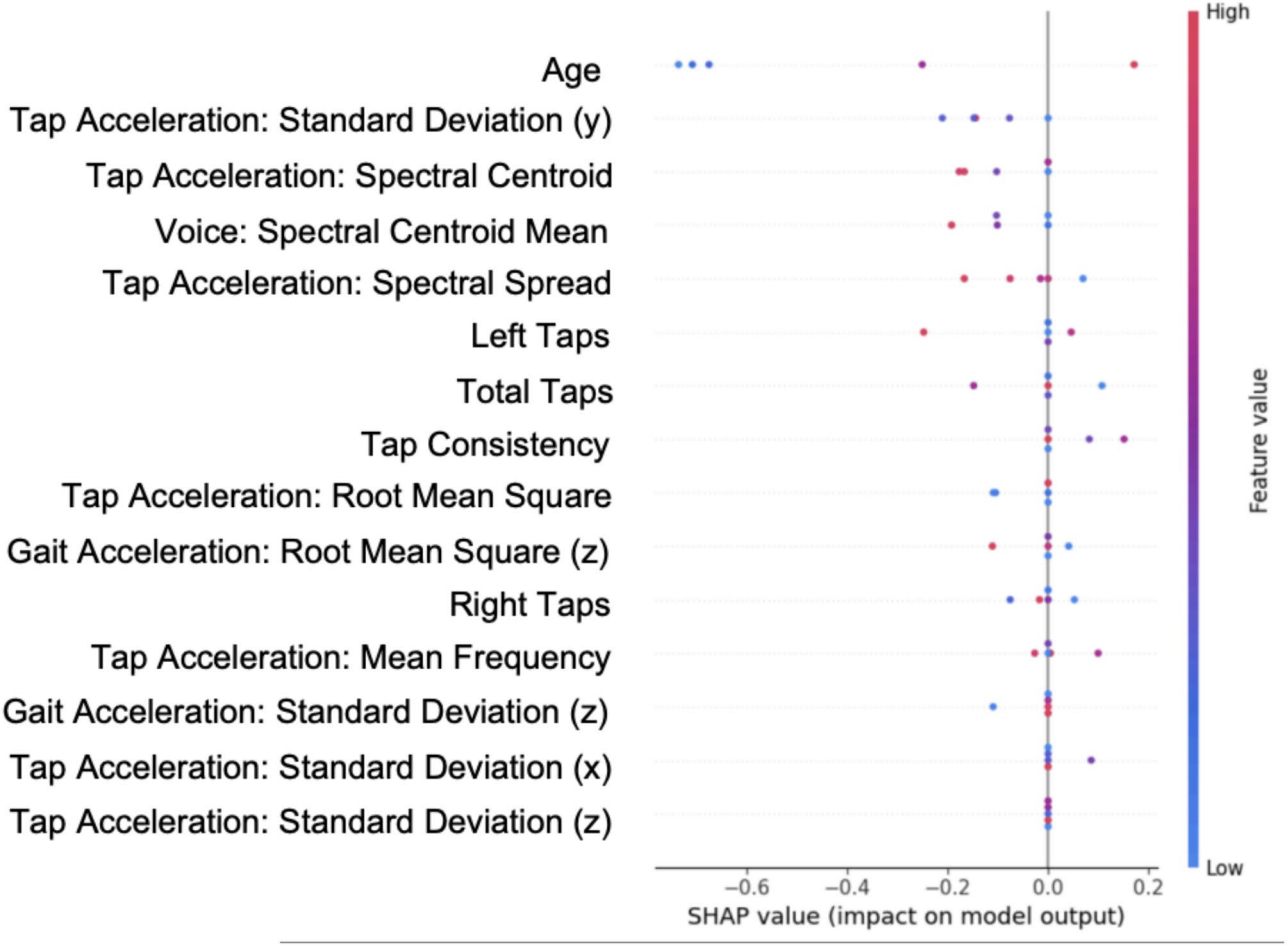
Feature importance in Parkinson’s disease detection model. SHAP values indicate feature impact on model predictions, with positive values (right) increasing disease probability and negative values (left) decreasing it. Color intensity represents relative feature importance.

Figure 3 displays the Shapley value plot, illustrating the relative importance of each feature in the model. As anticipated, age emerges as the most influential factor, exerting a significant effect across the predictive spectrum. Several tapping-related features—such as tap acceleration standard deviation (x, y, z), spectral centroid, and spectral spread—play significant roles, indicating that variability and frequency components of tapping movements contribute meaningfully to classification. Additionally, measures of tap consistency, total taps, and left/right taps show notable importance, reinforcing the relevance of motor coordination. Gait acceleration metrics, including root mean square (z) and standard deviation (z), further underscore the significance of movement-related biomarkers. Overall, these findings highlight the importance of motor-focused data, notably tapping dynamics and gait accelerations, as critical indicators for predicting Parkinson’s disease.

## Discussion

In this study, we employ a multimodal framework for prediction, using voice recordings with a universal vowel sound “ahh”, gait indications from phone-detected acceleration data points, phone tapping count and acceleration, as well as demographic information. By introducing a novel, simulatable quantum Support Vector Machine (qSVM) equipped with a custom kernel based on quantum rotation gates, we achieve high performance without requiring a fault-tolerant quantum device. This approach not only produces superior classification metrics but also demonstrates efficient and practical testing procedures, paving the way for more accessible clinical applications.

### Limitations

Concerns could arise regarding the model’s ability to generalize to new data. With only a train and test dataset, there is a possibility of the model overfitting to the validation set. Overfitting occurs when a model becomes overly complex, relying on the noise within the data rather than solely focusing on underlying patterns, causing the model to generalize poorly to real-world data^34^. Future studies could address this issue through rigorous clinical trials that expose the model to diverse patient populations, clinical environments, and real-world conditions, thereby offering a practical cross-validation of performance. Additionally, replicating the data collection procedures from the mPower study would help ensure consistency and robustness in subsequent applications.

### Ethics

All data used was made available by the mPower public researcher portal, and our academic usage complies with the guidelines outlined in the research license. In a real-world application, we acknowledge that false positives, though rare, could have the potential to cause psychological distress. Therefore, we assert that this model is not a standalone diagnostic but rather a screening tool, to be considered in the larger context of overall health by medical professionals. Furthermore, our work is intended to serve as a baseline for further research and testing. To enhance reliability and minimize errors, we propose a structured testing protocol similar to the measurements used in the mPower dataset.

Additionally, future studies should focus on evaluating the model across diverse populations and clinical settings, ensuring fairness and generalizability. Conducting controlled clinical trials will be essential in verifying the model’s effectiveness under real-world conditions, reducing biases, and refining its practical applications.

### Broader impact and future work

We aim to advance research in Parkinson’s disease (PD) prediction, with a particular focus on developing multimodal early screening tools that combine multiple biomarkers to enhance classification accuracy. Such noninvasive approaches boost the accessibility of initial classification while lowering costs compared to conventional methods. Moreover, incorporating a broad range of features not only improves model performance metrics but also facilitates a more individualized analysis, potentially enabling more tailored therapeutic strategies in future clinical settings. For example, future work could focus on broadening the spectrum of biomarkers by incorporating additional physiological and biochemical indicators, such as neuroimaging, genomic data, and other sensor-derived metrics.

We additionally encourage investigations into simulatable qSVM kernels for diverse real-world applications, as these may pave the way for new breakthroughs in various domains. The multimodal framework, which integrates various biomarkers and leverages advanced modeling techniques like qSVMs, can be readily extended to other neurological disorders characterized by complex symptom profiles. By adapting the feature extraction process to different physiological signals and clinical datasets, the approach holds promise for detecting a range of conditions beyond Parkinson’s disease, ultimately offering a flexible, powerful tool for broader healthcare applications.

For clinical deployment, such a model could be seamlessly integrated into existing healthcare infrastructures (e.g., EHRs) via digital platforms, allowing for remote and continuous patient monitoring. By embedding it into smartphone applications or wearable devices, physicians can more effectively track early symptoms and disease progression. Additionally, the model’s capacity to evaluate symptoms across multiple data modalities provides a holistic view of patient health, complementing conventional clinical evaluations. Healthcare providers can then leverage these consolidated insights directly within EHR platforms, enabling more personalized treatment strategies and streamlined patient monitoring.

In summary, we present a multimodal classifier for PD, trained on a diverse, globally representative dataset. This framework employs a quantum Support Vector Machine (qSVM) to integrate data from tapping measurements, gait tracking, voice recordings, and demographic information. To optimize resource utilization, the qSVM kernel relies on rotation gates rather than entanglement operations, permitting efficient classical simulations while preserving the strengths of quantum algorithmic principles. As a result, this design enables cost-effective and scalable PD prediction for a worldwide population.

Looking ahead, future work may prioritize conducting real-world clinical trials to gauge clinical utility, refining the model for improved interpretability, and fostering collaboration with healthcare providers for seamless integration into clinical practice.

## Methods

### Gait data processing

Although the mPower study included gait tracking measurements from a variety of different devices, including pedometers and accelerometers, we decided to focus on the smartphone measurements to preserve the accessibility and broad applicability of our model. The smartphone measurement file was composed of time series data tracking the x, y, and z acceleration of the device at regular intervals while the participant was walking. In order to interpret the data, we extracted the root mean square of the acceleration in the x, y, and z directions as well as that of the total signal, a metric commonly used to capture magnitude when describing time-varying quantities^35^. In addition, we included the standard deviation of acceleration in each direction to capture the variability over time. Next, we converted the total magnitude of acceleration into the frequency domain using a fast Fourier transform. In the frequency domain, we extracted the dominant frequency, mean frequency, spectral centroid, and spectral spread, corresponding to signal features that have the potential to capture the characteristic erratic movements of individuals affected by Parkinson’s disease^36^.

### Tapping feature extraction

In the tapping test, mPower participants used a smartphone app to alternate tapping a left button and a right button as many times as they could in 20 s. From this, two types of measurements were recorded. First, each left tap and right tap were recorded, along with their corresponding timestamps. From this information, we extracted the number of taps on the left button, right button, and the total number of taps in 20 s, which shows the dexterity of the participants. Next, we extracted the number of repeated taps on the same button, which shows the participant’s ability to alternate between buttons effectively. Finally, we extracted the consistency of their taps, which we measured by computing the standard deviation of the time between taps, corresponding to how consistent their tapping speed was.

The second type of measurement recorded was the acceleration of the smartphone in the x, y, and z directions throughout this 20 s interval. Similarly to gait, we extracted the root mean square and standard deviation of acceleration in the x, y and z directions, as well as for the total signal. In the frequency domain, we extracted the dominant and mean frequencies, in addition to the spectral centroid and spectral spread. Through all of the acceleration features, we hoped to represent the characteristic shakiness and tremors associated with PD^37^.

### Vocal feature extraction

While gait and demographic, and tapping data were expressed numerically, the researcher portal provided vocal data in the form of 10 s recordings of the vowel sound “ahhh”. To quantify the differences in voice, specific vocal characteristics were considered. Variations in volume and pitch have been known to be associated with PD^38^. As a result, their means and standard deviations were added to the dataframe. Furthermore, signal characteristics also capture variations in voice, so the means and standard deviations of the zero-crossing rate, root mean square, spectral centroid, spectral bandwidth, and spectral rolloff were included as well. Research has described Parkinson’s affected voices as having an airy or breathy quality^39^, and direct quantification of this feature was performed using the Acoustic Breathiness Index proposed by^40^. This index was added to the feature list, along with its inputs of harmonics to noise ratio, cepstral peak prominence, power spectral density, harmonic difference, glottal to noise excitation ratio, high-frequency noise occurrence at 6000 Hz, and shimmer in decibels. Finally, Mel Frequency Cepstral Coefficients were extracted for each recording, with Principal Component Analysis being applied to reduce the number of MFCC features to ten for computational efficiency.

### Demographic features

In addition to professional diagnosis, various information about the participants was provided. Excluding the hardware specifications, participant data mainly consisted of medical history. However, most of these measurements, such as past surgery, were not available for the majority of participants, hindering their efficacy. Furthermore, we excluded race to mitigate bias in detection. As such, age and smoking history were included due to a strong correlation with PD prevalence^41,42^, as well as gender because of its effect on vocal characteristics^43^. Table 3 provides further details on the demographic characteristics of the participants.

**Table 3:** Demographic breakdown of training and testing sets with significance for generalizability.

Demographic Attribute	Training set (*N* = 164)	Testing set (*N* = 30)	Total (*N* = 194)
Age distribution			
< 40 years	26	8	34
40–60 years	51	9	60
> 60 years	87	13	100
Race/Ethnicity			
Caucasian	138	26	164
People of color	26	4	30
Educational background			
Less than 4 years of college	53	8	61
4-year degree	39	10	49
Graduate Level/Higher Degree	70	12	82
Not reported	2	0	2
Smoker			
True	50	9	59
False	114	21	135

### *SVM* classifiers

Support vector machine (SVM) models are widely used in binary classification problems due to their flexibility across dataset sizes and resistance to overfitting^44^. By plotting each datapoint in high-dimensional space, the kernel function of an SVM seeks to define a hyperplane boundary between each class. In this way, the model is able to classify new data by plotting it on this same plane and seeing which side of the boundary it falls on. These boundaries, or kernels, can be linear, polynomial, or a radial basis function (RBF).

### *qSVM* model architecture

Quantum SVMs, or qSVMs, are being researched for their ability to improve classification by accessing high-dimensional Hilbert space. This works by considering the overlap or fidelity between quantum states, which has the potential to capture more complex relationships than a standard dot product. However, with current limitations on quantum hardware, quantum-inspired kernels that can be simulated on classical computers are more promising for real-world applications.

Currently, Qiskit offers the Fidelity Kernel constructor to construct quantum SVM models. This kernel mainly runs on the ZZ Feature Map^45^. This feature map has been known to achieve excellent performance on resource-intensive quantum hardware. However, due to its inherent complexity and reliance on entanglement, which can be difficult to simulate classically, this kernel performs poorly in classical state vector simulations, as supported by Simoes et al.^46^.

Past papers, such as Kariya et al. and Suzuki et al., have explored the use of specific rotational gates to encode data characteristics into a complex quantum state in conjunction with or as an alternative to entanglement^47,48^. This study proposes using Pennylane’s provided Angle Embedding function^30^, which encodes numerical data into rotation angles. This approach enhances the simplicity of the kernel construction by mitigating resource-intensive matrix operations or entanglement. The method encodes classical data as rotational angles on qubits, effectively transforming each input feature into a quantum state. By mapping numeric values to these rotations, Angle Embedding provides a straightforward yet powerful tool for simulating quantum-based machine-learning models on classical hardware. Furthermore, these rotations, represented as matrix operations applied to multidimensional quantum state vectors, have the potential to capture highly complex, nonlinear relationships.

### *qSVM* model framework

After each input is preprocessed and combined, the input data is mapped to a quantum feature space using our custom kernel. SVM kernels work by computing the similarity between two data points x_1_ and x_2_. To do this through quantum operations, each qubit represents one feature of the data. For each feature, the proposed model first performs a Y-axis rotation of the x_1_ value, followed by a Y-axis rotation of -x_2_. If x_1_ and x_2_ are similar, the resulting measurement would yield a value close to the qubit’s initial state of 0 since the rotations would nearly cancel out. In this way, by measuring the magnitude of the final qubit state, the kernel computes the overlap between x_1_ and x_2_.

**Fig. 4:**
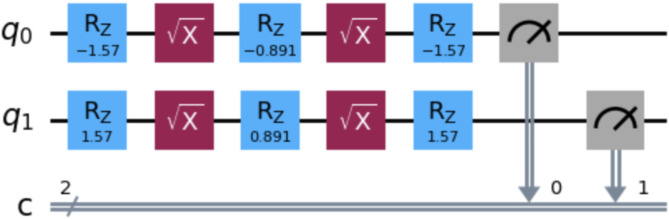
Example qubit encoding of one feature of the data. This qubit represents one of 15 qubits in the qSVM kernel circuit, with one qubit corresponding to each feature of the dataset. SVM kernels are designed to output a measure of similarity between two inputs x_1_ and x_2_. This figure shows the overlap computation between two inputs from the training set, using the gates R_Z_($\:-\frac{\pi\:}{2}$), $\:\sqrt{x}$, R_Z_(x_1_ - x_2_), $\:\sqrt{x}$ and R_Z_($\:-\frac{\pi\:}{2}$). These gates represent a quantum mechanical decomposition of R_Y_(x_1_) followed by R_Y_(-x_2_). Finally, the measurement gate at the end provides an output corresponding to the similarity between the inputs x_1_ and x_2_. Figure was generated using version 1.2.4 of the Qiskit Python library (https://pypi.org/project/qiskit/).

For ease of computation, the kernel replaces two rotations of R_Y_(x_1_) and R_Y_(-x_2_) with a single rotation of R_Y_(x_1_ − x_2_). As shown in Fig. 4, Qiskit breaks down R_Y_ operations into R_Z_ and R_X_ components, using quantum mechanical identities. Since R_X_($\:\frac{\pi\:}{2}$) is equivalent to $\:\sqrt{x}$, the kernel can be programmed as R_Z_($\:-\frac{\pi\:}{2}$), $\:\sqrt{x}$, R_Z_(x_1_ − x_2_), $\:\sqrt{x}$ and R_Z_($\:-\frac{\pi\:}{2}$), as shown. Since the rotation angles are entirely determined by the input data, no additional hyperparameters need to be tuned for this model.

**Fig. 5:**
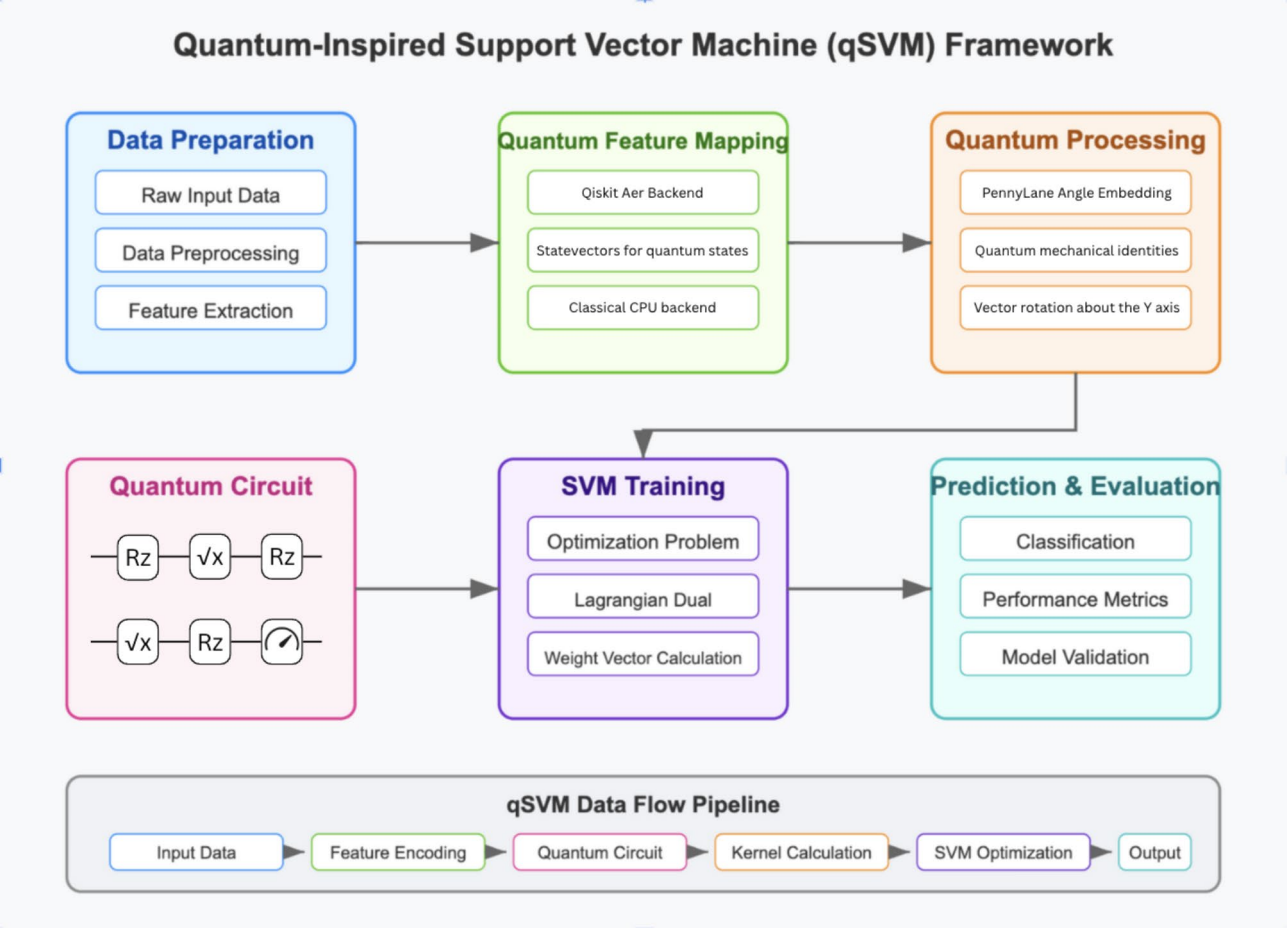
Quantum-Inspired Support Vector Machine (qSVM) Framework. The diagram outlines a qSVM architecture where classical data is processed through a hybrid quantum-classical pipeline: *Data Preparation* Raw data is cleaned, normalized, and features are extracted for quantum encoding. Quantum Feature Mapping: Classical data is embedded into a quantum feature space using statevector simulation. Quantum Processing: Quantum kernels, measuring data similarity in the quantum space, are computed. Quantum Circuit: Quantum operations on qubits are executed, including the decomposition of the RY gate into RZ and √x gates. SVM Training: A classical optimizer employs the quantum kernel matrix to determine the optimal separating hyperplane. Prediction & Evaluation: New data is classified and performance is evaluated using metrics such as accuracy, precision, and recall. The pipeline follows the sequence: Input Data → Feature Encoding → Quantum Circuit → Kernel Calculation → SVM Optimization → Classification. This approach integrates quantum-enhanced feature representation with traditional SVM for improved classification.

After each qubit is measured, the values are aggregated through a weighted sum of each measurement multiplied by the random forest feature importance of the corresponding feature, transformed using a softmax function to emphasize the contribution of features with high predictive power. This computation is repeated on every pair of datapoints in the training set to construct the kernel matrix, which is then passed into a classical SVM for evaluation as shown in the schematic diagram for the quantum-inspired framework and data flow (Fig. 5).

The proposed model mitigates overfitting by first selecting only the most predictive features using a Random Forest (above the 80th percentile) and scaling them by importance, and by employing a simplified kernel based on quantum rotation gates. Additionally, using a single trial per participant prevents individual data from dominating the training set, further enhancing generalizability.

### Benchmark models

All benchmark models were chosen from either standard machine learning options or models used by prior researchers in this field^22,28,29,31^. For most models, the same training and testing sets as the proposed model were used to ensure a fair comparison. However, for the alternative qSVM kernels of the Z and ZZ feature map, the full dataset was too resource-intensive to run. So, we chose to extract metrics based on a subset of the dataset including the first 30 train and 15 test samples.

## Electronic supplementary material

Below is the link to the electronic supplementary material.

Supplementary Material 1

## Data Availability

The datasets generated and/or analysed during the current study are available in the mPower Public Research Portal^23^ repository, https://www.synapse.org/Synapse:syn4993293/wiki/376006. This dataset is publicly available at the link noted.
