# Supplementary material for: A quantum inspired machine learning approach for multimodal Parkinson’s disease screening
Source: Sci Rep. 2025 Apr 4;15:11660. doi: 10.1038/s41598-025-95315-0 (PMC11971407; doi:10.1038/s41598-025-95315-0)
Supplement: Supplementary file 1 — Supplementary Material 1 [file 41598_2025_95315_MOESM1_ESM.docx]

**Dataset Access**

All models referenced in the paper were trained on the mPower Public Research Portal (<https://www.synapse.org/Synapse:syn4993293/wiki/376006>), a collection of various measurements corresponding to biomarkers commonly associated with Parkinson’s disease, as well as demographic information for each participant. These tests include a voice recording, tapping test and gait acceleration measurement.

Although the dataset is publicly available, the data is under protected access. Samples are available but to access diagnosis labels and demographic information, a certification is needed ensuring safe use of participants’ personal information.

After obtaining access, the instructions for downloading the data programmatically are included in the Synapse documentation (<https://python-docs.synapse.org/tutorials/home/>). To mitigate bias toward participants who completed the same test multiple times, we chose to include the first such test for each participant. Then, we only included the participants who had participated in all diagnostic tests in order to build a multimodal predictor. This process yielded 234 suitable participants. Finally, we dropped the first 40 participants who did not have Parkinson’s disease in order to balance the dataset, yielding 194 participants, equally split between the healthy and affected classes.

**Code Replicability**

All code is included in our GitHub repository (<https://github.com>). Among these, “data_processing.ipynb” was used first to extract features, “feature_selection.ipynb” was used next to extract the features above the 80th percentile, “proposed_model.ipynb” was used afterwards to train the focal classification model and “benchmark_models.ipynb” was used last to compare the performance of the proposed model against multiple standard models.
